# Estimating the impact of interventions on cause-specific maternal mortality: a Delphi approach

**DOI:** 10.1186/1471-2458-13-S3-S12

**Published:** 2013-09-17

**Authors:** Suzanne Lee Pollard, Matthews Mathai, Neff Walker

**Affiliations:** 1 Department of International Health, Johns Hopkins Bloomberg School of Public Health, 615 N. Wolfe St., Baltimore, MD 21205, USA; 2 Department of Maternal, Newborn, Child and Adolescent Health, The World Health Organization, Geneva 1211, Switzerland

## Abstract

**Background:**

Approximately 287,000 women die of causes related to pregnancy and childbirth every year. While effective interventions exist to prevent maternal death, high quality impact evaluations for these interventions are often lacking.

**Methods:**

We conducted a Delphi process consisting of three rounds in which we asked maternal health experts to provide effectiveness estimates for 31 intervention-cause of death pairs relating to maternal mortality. Anonymous feedback in the form of medians and histograms for each question was given to experts following the first and second rounds. A diverse panel of 37 experts completed all three rounds, for a final response rate 80.4%.

**Results:**

This Delphi process produced a total of 31 effectiveness estimates for key maternal interventions on cause-specific maternal mortality. Overall, many interventions had high estimated effectiveness, with the majority of interventions having effectiveness estimates above 70%. Where possible, the estimates of effectiveness of interventions were compared to previous efforts and in general there was strong agreement between the estimates in this exercise as compared to those of earlier efforts.

**Conclusions:**

There are many maternal health interventions with high estimated effectiveness that, with expansion of effective delivery channels, have the potential to have a large impact on reducing maternal mortality worldwide.

## Background

Every year, approximately 287,000 women die of causes related to pregnancy and childbirth worldwide, with nearly all of those deaths occurring in low- and middle-income countries [1]. While effective interventions and packages exist to prevent maternal death, high-quality impact evaluations generated from randomized trials or large observational studies are often lacking due to the large sample sizes required to detect statistical differences in mortality. In addition, some maternal interventions that have been routine practice for decades, such as caesarean sections, will never be studied through randomized trials due to ethical considerations. Furthermore, the effects of specific interventions normally found within packages are difficult to assess in experimental studies. For such interventions, it is necessary to obtain effectiveness estimates through other methods.

We set out to conduct this Delphi analysis to generate the best estimates for specific interventions on causes of maternal death, and to use these estimates to update the maternal model in the Lives Saved Tool (LiST) in light of the World Health Organization (WHO)’s new cause of death structure for maternal mortality [2]. LiST is a computer-based model that estimates the impact of scaling up interventions on maternal and child mortality, along with other health outcomes (e.g., stunting rates, stillbirths, diarrhea incidence) to help guide program planning [3]. The development of the LiST model has been directed by the Child Health Epidemiology Reference Group (CHERG) of WHO and the United Nations Children’s Fund (UNICEF). This group has developed a set of standards by which interventions should be included in the model and how estimates of intervention effectiveness should be developed. For interventions where there is clear clinical/preventive benefit of an intervention but no data available, a Delphi approach could be used to generate estimates of the effectiveness of these interventions [4,5].

The Delphi method, developed by the RAND Corporation in 1948, is a method by which consensus is achieved through an iterative process by a panel of experts. The method was originally developed in defense research for scientific and technological forecasting purposes. Essential components of the Delphi method include anonymous response, iteration and controlled feedback, and a statistical definition of consensus [6]. It has been used historically in a variety of disciplines, and has been applied to a variety of areas within the field of health and medicine [7-10]. The Delphi method is thus a widely-accepted and useful tool for generating estimates in the absence of sufficient evidence from experimental studies.

Previously, WHO sponsored a Delphi exercise to estimate the impact of interventions on maternal and neonatal mortality [unpublished manuscript, WHO]. The purpose of WHO’s previous Delphi analysis was to identify areas of consensus and disagreement regarding the efficacy of newborn and maternal health interventions, with the ultimate goal of evaluating the cost-effectiveness of these interventions [unpublished manuscript, WHO]. The estimates of effectiveness along with others generated within the CHERG framework [11] were used for the estimates of effectiveness in LiST. Recently, WHO redefined the categories within cause-specific maternal mortality, and therefore the estimates of effectiveness needed to be updated to reflect this new set of mortality causes.

In this paper, we describe the process by which these consensus estimates were generated and discuss the key findings of this Delphi analysis. In addition, we outline how these results will be incorporated into the maternal model in LiST. It is important to note that this study does not constitute a literature review or meta-analysis, but rather presents a series of effectiveness estimates generated through an iterative process involving a group of experts in maternal health.

## Methods

### Participants

We identified 90 international experts in maternal health and contacted them by email for participation in this study. The original set of experts we contacted was selected by identifying researchers who had worked with the CHERG and asking those people to suggest other participants. The pool also included some experts in maternal health who had participated in a prior Delphi panel with WHO. We tried to ensure diversity in professional experience and in geographic background, and to achieve a mix of experts in public health research and clinical work, and those with familiarity or experience in global health.

Of the 90 experts we contacted, 46 completed the initial questionnaire. The response rate for round one was 100% by definition. Four experts declined to participate, and 40 did not respond. 40 of the 46 experts who completed the round one questionnaire completed round two, for a response rate of 87%. The final response rate was 82.6%, with 38 out of the original 46 experts completing the third round. One of these 38 experts completed the third round, but did not complete the second round.

Of the experts who completed the round one questionnaire, a slight majority were female. Expert panel members represented 22 different nationalities and worked in every region of the globe.

The expert panel was comprised primarily of obstetricians and researchers, with nurse/midwives also being represented. In addition to being physicians and nurses, 11 clinical experts identified themselves as researchers, and three identified education as a secondary profession. A majority of the experts had medical degrees, and many had more than one degree (see Table 1).

**Table 1:** Demographic characteristics of the expert panel

**Sex**	**Number**	**Percentage**
Male	22	47.8
Female	24	52.2

**Region**	**Nationality, n (%)**	Experts working in each region, n (%)*
Europe	10 (21.7)	21 (7.1)
Americas		
*U.S./Canada*	9 (19.6)	3 (4.3)
*Latin America*	4 (8.7)	10 (8.6)
Southeast Asia	8 (17.4)	7 (25.7)
Western Pacific	7 (15.2)	5 (14.3)
Africa	5 (10.9)	18 (30)
Eastern Mediterranean	3 (6.5)	6 (10)
**Total**	46 (100)	70 (100)

**Profession**	**n (%)**	
Obstetrician/Gynecologist	30 (65.2)	
Researcher/Academic/ Epidemiologist	8 (17.3)	
Nurse/midwife	5 (10.9)	
Other physician	2 (4.3)	
Public health practitioner	1 (2.2)	
**Total**	46 (100)	
**Professional experience**	**Median**	
Median years working in profession	30	
**Academic Qualifications**	**n (%)**	
Doctorate	5 (10.9)	
Master’s Degree	2 (4.4)	
Medical Degree plus further qualification	19 (41.3)	
Medical degree	16 (34.8)	
Nursing degree plus further qualification	3 (6.52)	
Nursing degree	1 (2.17)	
**Total**	46 (100)	

*The total for this column is greater than number of experts, as some experts work in multiple regions.

### Materials

We developed a questionnaire in which experts were asked to provide estimates for the effectiveness of the specified intervention on a specific cause of maternal death. We developed the questions through consultation and pilot testing with several maternal health experts over a six-month period. We began the process with 23 individual interventions and three packages coming primarily from LiST’s current maternal and neonatal health models. We identified seven categories of causes of maternal death based on the most recent cause of maternal death structure developed by WHO [2]. Initially, all possible intervention-cause of death pairs were included; throughout the piloting phase, we excluded questions that were likely to produce negligible effects on the prevention of maternal deaths. The final questionnaire had a total of 31 questions.

The questions were framed in such a way that experts were asked to provide effectiveness estimates for the intervention on maternal deaths due to a specific cause. For example, we asked for the effectiveness of calcium supplementation on maternal deaths due to pre-eclampsia/eclampsia. We specified in the instructions that interventions were assumed to be timely and of high quality. The experts had the option to complete the questionnaire through an online form or to complete a Word document version of the questionnaire and send through email.

### Procedures

#### Rounds and group feedback

We decided *a priori* to run three rounds of this questionnaire. Three rounds are generally accepted as appropriate to balance attrition rate and fatigue against conducting a sufficient number of rounds to reach consensus [12]. The purpose of the initial round was for experts to provide their initial estimates for each of 31 intervention-cause of death pairs. The second and third rounds were to allow the experts to revise their responses based on group feedback. In the second round, experts were provided with their individual responses to round one, the median of the group responses, and a histogram of group responses for each question. In the third round, experts were provided with individual responses, medians, and histograms for round two; if desired, experts could also refer to the responses from round one. We hoped that by providing the full range of responses in a histogram instead of just providing feedback in the form of a mean or median value, we would reduce the pressure on individual respondents to conform to median values. All 31 questions were included in the each round regardless of whether consensus had been achieved in previous rounds.

#### Definition of consensus

Consensus was defined to be an interquartile range (IQR) of 20 percentage points or fewer. The median response was used as the final effectiveness estimate.

## Results

We first conducted an analysis to characterize changes in effectiveness estimates and the degree of consensus across rounds. We then present the resulting effectiveness estimates for each intervention-cause of death pair across all rounds.

### Changes in effectiveness estimates and degree of consensus across rounds

The median change in response for all responses for all questions was 0 [IQR: -5,5] percentage points from round one to round two, ranging from -85 to 78. The median change was 0 [IQR: 0,0] percentage points from round two to round three, ranging from -53 to 95. The IQRs for all intervention-cause of death pairs diminished from round to round. The IQRs in round one ranged from five to 63.75, with a median IQR of 36.25 [IQR: 25, 43.75]. The IQRs in round two ranged from five to 41.25, with a median of 20 [IQR: 14.25, 26.25]. In round three, the median IQR of all the pairs was 15 [IQR: 10, 20], ranging from 3.75 to 30.

### Effectiveness values by round and final effectiveness values

The effectiveness estimates and IQRs for the final round are presented in Table 2. Many interventions had high estimated effectiveness, with 18 out of 31 intervention–cause of death pairs having 70% estimated effectiveness or higher. Interventions to prevent maternal deaths due to hypertensive disorders of pregnancy (HDPs) had effectiveness estimates of 50% or less, except for interventions that included MgSO_4_ (anti-convulsants: 60% effectiveness, Basic Emergency Obstetric Care [BEmOC]: 60% effectiveness, Comprehensive Emergency Obstetric Care [CEmOC]: 85% effectiveness). Safe abortion services and post-abortion care were both estimated to be highly effective against deaths due to abortion (95% and 80% effectiveness, respectively), and ectopic pregnancy case management had a comparably high estimated effectiveness against ectopic pregnancy deaths (90% effectiveness). Parenteral antibiotics and packages that include them (BEmOC, CEmOC) were more effective than clean delivery practices or blood transfusion against deaths due to pregnancy-related sepsis according to our estimates (see Table 2).

**Table 2:** Effectiveness estimates and interquartile ranges for maternal interventions on cause-specific maternal mortality

**Intervention**	**Cause of maternal death**	**Final estimate [IQR]**	**Intervention**	**Cause of maternal death**	**Final estimate [IQR]**
Calcium supplementation	Pre-eclampsia / eclampsia	*20 [15,30]	BEmOC	APH	**40 [25,50]**
Anti-convulsants (MgSO_4_)	Pre-eclampsia / eclampsia	*60 [50,70]	CEmOC	APH	*90 [90,95]
BEmOC	Pre-eclampsia / eclampsia	*60 [50,70]	Assisted vaginal delivery	Prolonged and obstructed labor	*38.8 [30,45]
CEmOC	Pre-eclampsia / eclampsia	*85 [80,90]	Caesarean section	Obstructed labor	*90 [85,90]
Hypertensive therapy	HDPs	**50 [30,60]**	Blood transfusion	Obstructed labor	*25 [20,30]
Induction of labor	HDPs	**42.5 [25,50]**	CEmOC	Obstructed labor	*95 [90,95]
Caesarean section	HDPs	*45 [30,50]	Uterotonics (prevention)	PPH	*77.5 [60,80]
Safe abortion services	Induced abortion	*95 [90,95]	Uterotonics (treatment)	PPH	*80 [70,90]
Post-abortion care	Induced abortion	*80 [70,90]	AMTSL	PPH	*70 [60,80]
Blood transfusion	Abortions and ectopic pregnancy	*60 [50,70]	Manual removal of the placenta	PPH	*30 [20,30]
Ectopic pregnancy case management	Ectopic pregnancy	*90 [85,95]	BEmOC	PPH	*75 [60,80]
Clean delivery practices	Sepsis-pregnancy	**60 [50,75]**	CEmOC	PPH	*90 [90,95]
Parenteral antibiotics	Sepsis-pregnancy	*80 [70,85]	ARVs	HIV/AIDS	*70 [60,80]
Blood transfusion	Sepsis-pregnancy	*25 [20,30]	IPTp/ITNs	Malaria (*P. falciparum*)	*72.5 [70,80]
BEmOC	Sepsis-pregnancy	*70 [60,80]	Malaria case management	Malaria (*P. falciparum*)	*80 [75,90]
CEmOC	Sepsis-pregnancy	*90 [80,90]			

*IQR ≤ 20 percentage points

CEmOC was estimated to be highly effective against deaths due to antepartum hemorrhage (APH) (90% effectiveness) as compared to BEmOC (40% effectiveness). Caesarean section and CEmOC had the highest estimated effectiveness against obstructed labor deaths (effectiveness 90% and 95%, respectively), and packages of interventions such as active management of the third stage of labor (AMTSL) (70% effectiveness), BEmOC (75%), and CEmOC (90%) were highly effective against deaths due to postpartum hemorrhage (PPH). Finally, intermittent preventive treatment in pregnancy (IPTp)/insecticide-treated nets (ITNs) and malaria case management had moderately high effectiveness estimates against malaria-specific maternal deaths (72.5% and 80%, respectively), and adult antiretrovirals (ARVs) had an effectiveness estimate of 70% against maternal deaths due to HIV. Interestingly, CEmOC not only had high estimated effectiveness against five causes of maternal death (pre-eclampsia/eclampsia, pregnancy-related sepsis, APH, obstructed labor, and PPH), but the uncertainty for the effectiveness of CEmOC against these causes of death was also low, with IQRs of 10 or narrower for all five estimates.

## Discussion

Overall, most interventions showed high estimated effectiveness, suggesting that there are many highly-effective interventions to prevent maternal death. Even interventions such as MgSO_4_, which can be delivered at small community health centers as opposed to hospitals, are estimated to be very effective against maternal death due to a variety of causes. These results suggest that the next step in combating maternal mortality may be to develop better ways to deliver these interventions to communities that are the most in need, as opposed to developing and testing new interventions. With regard to group agreement, the IQRs for nearly all intervention-cause of death pairs decreased across rounds, indicating consistent movement toward consensus. By the final round, a total of four intervention-cause of death pairs had an IQR of effectiveness estimates greater than 20 percentage points, although none of these had an IQR greater than 30 percentage points. Two of these pairs were related to HDPs and are discussed later. One of these pairs asked for the effectiveness of clean delivery practices on maternal deaths due to pregnancy-related sepsis. As clean delivery practices are an intervention more often associated with newborn health, experts may have been less familiar or confident in their ability to assess the impact of this intervention on maternal mortality. It is also interesting to note that while the IQR of the estimates of effectiveness did get smaller over the rounds, the mean and median point estimates did not change by a large degree for most intervention-outcome pairs.

There are several strengths to our study that likely enhance the validity of our results. First, a diverse panel of experts participated in this Delphi process, representing multiple professions, 22 different nationalities, and working in many countries in all regions of the world. This diversity promotes a more predictive and valid consensus in studies related to health [13]. Furthermore, we rigorously pilot-tested our questionnaire with multiple maternal health experts prior to the actual Delphi process in order to ensure that questions and definitions were as clear and complete as possible. Another important advantage to our approach was the format of the feedback provided to our experts. While many Delphi processes may provide only median values or IQRs as post-round feedback, our experts were provided with median values in addition to a histogram with all responses from the previous round. This form of feedback allowed experts to consider the full range of responses and to better assess group consensus as they provided their revised answers.

The estimates generated from this Delphi analysis, in conjunction with estimates generated from other sources, will be used in the maternal mortality model in LiST. Each intervention-cause of death pair and the corresponding effectiveness estimate from this Delphi process will represent a link in the model, and all pairs described in this paper will be included in the final model. We will structure the model using the most recent WHO maternal cause of death categories as a framework [2]. In instances where we asked for the effectiveness of an intervention on a specific cause of death, we will use affected fractions representing the proportion of maternal deaths within the given WHO category represented by that specific cause. For example, pre-eclampsia and eclampsia represent a given proportion of maternal deaths due to HDPs. To determine the effectiveness of MgSO_4_ on deaths due to HDPs, this affected fraction will be multiplied by the effectiveness of that intervention on pre-eclampsia/eclampsia in order to determine the number of maternal deaths prevented within the larger category. In addition, in the case of packages such as BEmOC, LiST users will have the option to scale up certain individual interventions within the package or to scale up the entire intervention package as a unit.

Several of the interventions in the Delphi were also included in a 2011 review by Ronsmans and Campbell [11]. This review discusses five interventions that were included in our Delphi analysis: calcium supplementation, MgSO_4_, and CEmOC for treatment of pre-eclampsia and eclampsia, and hypertensive drugs and induction of labor for the treatment of HDPs. The results of this review are largely consistent with the results of our Delphi process (see Table 3). Ronsmans and Campbell report that calcium supplementation can reduce death or serious morbidity due to HDPs by 20%, which is the same as our consensus estimate for the effect of calcium supplementation on deaths due to pre-eclampsia and eclampsia. In addition, they report a 41% reduction in maternal death for those treated with MgSO4 as compared to diazepam. Our consensus effectiveness estimate was 60%, which may be reasonable given that our questionnaire asked for an estimate in comparison to the absence of treatment. Ronsmans and Campbell also report a 84-99% reduction in mortality due to severe pre-eclampsia and eclampsia for a package that includes antenatal screenings, MgSO_4_, and early delivery. This estimate is consistent with our effectiveness estimate of 85% for CEmOC on pre-eclampsia and eclampsia. This review may also shed light on why two interventions related to HDPs in our Delphi analysis, antihypertensive therapy and induction of labor, had relatively large IQRs (greater than 20 percentage points) after the final round. Ronsmans and Campbell report that while antihypertensive drugs halve the risk of developing severe hypertension, their effect on mortality is unclear due to lack of quality evidence. In addition, they conclude that while induction of labor is effective in reducing adverse maternal outcomes, it is impossible to assess the consistency of the effect of induction of labor on HDP mortality based on current evidence [11].

**Table 3:** Comparison of Delphi effectiveness estimates with estimates from Ronsmans and Campbell 2011 [11]

LiST Delphi Study			**Ronsmans & Campbell (2011) **[11]		
*Intervention*	*Cause of maternal death*	*Estimate*	*Intervention*	*Cause of maternal death*	*Estimate*

Calcium supplementation	Pre-eclampsia / eclampsia	20	Calcium supplementation in pregnancy	Maternal death or serious morbidity due to HDPs	20

Anti-convulsants (MgSO4)	Pre-eclampsia / eclampsia	60	MgSO_4_ (as compared to diazepam)	Maternal death (unspecified)	41

Hypertensive therapy	HDPs	50^§^	Antihypertensives	HDPs	Inconclusive

Induction of labor	HDPs	42.5^§^	Induction of labor	HDPs	Inconclusive

CEmOC	Pre-eclampsia / eclampsia	85	Antenatal screening for hypertension and proteinuria and treatment of pre-eclampsia and eclampsia with MgS0_4_ and early delivery in women with severe preeclampsia and eclampsia	Severe pre-eclampsia and eclampsia	84-99

^§ ^**=** consensus not reached

In 2006, WHO conducted a Delphi process to generate estimates for the efficacy of interventions to reduce maternal and neonatal mortality [unpublished manuscript, WHO]. While several factors limit our ability to directly compare these two analyses, the results of these two Delphi processes are overall consistent and confirmatory (see Table 4). For example, in our Delphi, the estimated effectiveness of safe abortion services on maternal death due to induced abortion was 95%, while the efficacy estimate of a similar intervention in the WHO Delphi (“management of abortion complications to protect mothers with complications of abortion”) on complications of abortion was 90%. Any discrepancies found between our estimates and those of the WHO Delphi can be explained by differences in the definitions and delimitations of interventions and causes of death. In our Delphi process, for example, the estimated effectiveness of clean delivery practices on pregnancy-related sepsis was 60%, as opposed to 75% in the WHO Delphi. However, the WHO Delphi specifically asked for the efficacy on one cause of sepsis, puerperal metritis. Thus, the difference in estimates may be explained by the fact that clean delivery practices are considered most effective against puerperal sepsis, and less so against other causes of sepsis, which were included in our definition. Hence, there were no large discrepancies between the results of our Delphi and those of WHO’s that could not be explained by differences in definitions. Also of interest is that overall, the uncertainty of estimates in the WHO Delphi after three rounds were a few points narrower than ours, given that the WHO defined a consensus estimate as having an IQR of 15 percentage points or fewer. One likely explanation for this difference is the form of post-round feedback that was provided to the experts. While our feedback included histograms of all responses to the previous round, the WHO Delphi only used median values as a form of feedback, which may have led to stronger pressure to move toward the median values on subsequent rounds.

**Table 4:** Comparison of Delphi effectiveness estimates to WHO Delphi (2006)

LiST Delphi Study			WHO Delphi Study (2006)			
*Intervention*	*COD*	*Est.*	*Intervention*	*COD*	*Est.*	*Notes*

CEmOC	PPH	90	Management of PPH to protect mothers with PPH more than 500 ml after birth	PPH	90	WHO intervention could be carried out in CEmOC facility
Safe abortion services	Induced abortion	95	Safe abortion, Management of abortion complications to protect mothers with complications of abortion	Complications of abortion	90	
CEmOC	Obstructed Labor	95	Management of prolonged or obstructed labor to protect mothers with prolonged or obstructed labor	Obstructed labor	90	WHO intervention could be carried out in CEmOC facility
Parenteral antibiotics	Sepsis-pregnancy	80	Management of puerperal sepsis to protect mothers with puerperal sepsis	Puerperal metritis	80	Puerperal metritis is only one cause of pregnancy-related sepsis
IPTp/ITNs	Malaria	72.5	Malaria prophylaxis or IPT provided during the antenatal period in malaria endemic areas	Malaria	80	WHO intervention does not include ITNs
IPTp/ITNs	Malaria	72.5	ITNs	Malaria	24^§^	WHO intervention does not include IPTp
Anti-convulsants (MgSO4)	Pre-eclampsia/eclampsia	60	Treatment of HDPs	Pre-eclampsia or eclampsia	80	WHO intervention also includes anti-hypertensives and supportive care
Hypertensive therapy	HDPs	^50§^	Treatment of HDPs	Pre-eclampsia or eclampsia	80	WHO intervention includes MgSO4 and supportive care in addition to antihypertensives
CEmOC	APH	90	Management of APH to protect mothers with APH	APH	80	WHO intervention could be carried out in CEmOC facility
Clean delivery practices	Sepsis-pregnancy	60^§^	Clean delivery practices by traditional birth attendant	Puerperal metritis	75	Puerperal metritis is only one cause of preg.-rel. sepsis
Parenteral antibiotics	Sepsis-pregnancy	80	Management of pre-labor rupture of membranes and infection to protect mothers with pre-labor rupture of membranes and infection	Puerperal metritis	55	Puerperal metritis is only one cause of preg.-rel. sepsis; WHO intervention is only preventive
Parenteral antibiotics	Sepsis-pregnancy	80	Antibiotics for preterm premature rupture of membranes	Puerperal metritis	50	Puerperal metritis is only one cause of preg-rel. sepsis; WHO intervention is only preventive

^§ ^**=** consensus not reached, COD = cause of death

The different definitions and categories are due largely to our use of the new WHO maternal cause of death structure. It is important to note that the WHO process was conducted nearly six years prior to ours [unpublished manuscript, WHO]. In addition, the WHO Delphi questionnaire asked for efficacy estimates as opposed to effectiveness estimates, although the two are often conflated, and in our survey we specified that interventions were timely and of high quality. Nevertheless, it is useful to compare our findings against another comparable review, which included a different panel of experts and thus a somewhat different mix of nationalities and professions.

There were two inconsistencies in our results that are worth noting. First, effectiveness estimates for uterotonics on both prevention and treatment of PPH deaths were higher (77.5% and 80%, respectively) than that for AMTSL (70%). AMTSL is a package that includes uterotonics, controlled cord traction, and manual removal of the placenta; thus, we would expect that AMTSL would have the same or higher effectiveness on PPH than uterotonics in isolation. Similarly, the effectiveness estimate for parenteral antibiotics, a component of BEmOC, on pregnancy-related sepsis, was higher (80%) than that for BEmOC (70%). While these inconsistencies are important to be aware of, in both cases, the differing effectiveness estimates are within their respective limits of uncertainty. For this reason, we have chosen to include these pairs in our model despite the inconsistencies. Furthermore, the fact that we were able to identify these inconsistencies shows a benefit to our approach in that had we only asked about packages as a whole, these differences would not have been identifiable. In order to handle these inconsistencies in the model, we will use the higher effectiveness value of the individual component to represent both the effectiveness of that component and the package contained within.

There are several limitations to our methodology. An important limitation of the Delphi method lies in the method for defining consensus. While there has been considerable discussion surrounding this issue, there seems to be little agreement as to how to define consensus [14]. Thus, the definition of consensus is inherently determined at least partially by the subjective opinion of the researcher. While we chose to define consensus as an IQR of 20 percentage points or fewer, we recognize that this number is somewhat arbitrary and useful primarily as an analytical tool. As such, all estimates that were produced in this Delphi analysis will be included in the final LiST model regardless of whether or not consensus was reached, with corresponding uncertainty values included. Another important limitation relates to the way in which each individual expert interprets each question. While we piloted the questionnaire with multiple maternal health experts prior to beginning the Delphi process, there is inevitable variation in interpretation. In addition, although panel members had considerable experience and expertise in their field, the Delphi method is inherently based partially on the opinions and biases of individual experts. For this reason, we sought out a heterogeneous panel that would represent a diversity of responses and opinions. However, our results, as well as which interventions are included in the model, are influenced by “hot topics” and current opinions in the global health field and will inevitably evolve as the field evolves. Furthermore, although all experts were provided with a Word document with their responses to previous rounds in addition to group responses, the online questionnaire itself did not display individual responses. If those experts who completed the questionnaire online were less aware of their own responses than those who responded using the Word document, this may have influenced their responses to the subsequent round. Despite these limitations, the Delphi method can be a powerful tool in situations where the evidence base is lacking or where it is not feasible to perform large-scale observational studies and randomized trials due to ethical considerations. In this case, this method has enabled us to use expert opinion to generate estimates that will allow for the prioritization of public health programming.

The maternal mortality and other models used in LiST are constantly evolving, requiring periodic updating to incorporate new and emerging evidence. In addition, it may be important to include certain risk factors such as anemia in future maternal models, which may impact maternal survival by affecting multiple causes of maternal death simultaneously.

## Conclusions

The results of this Delphi process suggest that there are many existing maternal health interventions with high estimated effectiveness. With the expansion of effective delivery channels, existing interventions have the potential to have a large impact on reducing maternal mortality worldwide.

## List of abbreviations used

AMTSL: Active Management of the Third Stage of Labor; APH: Antepartum hemorrhage; ARVs: Antiretrovirals; BEmOC: Basic Emergency Obstetric Care; COD: Cause of death; CHERG: Child Health Epidemiology Reference Group; CEmOC: Comprehensive Emergency Obstetric Care; HDP: Hypertensive disorders/diseases of pregnancy; ITNs: Insecticide-treated nets; IPTp: Intermittent preventive treatment in pregnancy; IQR: Interquartile range; LiST: Lives Saved Tool; PPH: Postpartum hemorrhage; UNICEF: United Nations Children’s Fund; WHO: World Health Organization.

## Competing interests

The authors have no competing interests to declare.

## Authors' contributions

SLP participated in the design of the study, coordinated the Delphi process, conducted the analyses, and drafted the manuscript. MM participated in the design and coordination of the study. NW conceived of the study, participated in its design and coordination, and helped to draft the manuscript. All authors read and approved the final manuscript.

## Declarations

The participation of Matthews Mathai and the feedback from various staff members at WHO are not to be taken as approval of or agreement with the findings from the World Health Organization. The findings in this paper are those of the three authors only.

The publication costs for this supplement were funded by a grant from the Bill & Melinda Gates Foundation to the US Fund for UNICEF (grant 43386 to "Promote evidence-based decision making in designing maternal, neonatal, and child health interventions in low- and middle-income countries”). The Supplement Editor is the principal investigator and lead in the development of the Lives Saved Tool (LiST), supported by grant 43386. He declares that he has no competing interests.

This article has been published as part of *BMC Public Health* Volume 13 Supplement 3, 2013: The Lives Saved Tool in 2013: new capabilities and applications. The full contents of the supplement are available online at http://www.biomedcentral.com/bmcpublichealth/supplements/13/S3.
